# Impacts of Tropical Cyclones and Accompanying Precipitation on Infectious Diarrhea in Cyclone Landing Areas of Zhejiang Province, China

**DOI:** 10.3390/ijerph120201054

**Published:** 2015-01-22

**Authors:** Zhengyi Deng, Huanmiao Xun, Maigeng Zhou, Baofa Jiang, Songwang Wang, Qing Guo, Wei Wang, Ruihua Kang, Xin Wang, Gifty Marley, Wei Ma

**Affiliations:** 1 Department of Epidemiology and Health Statistics, School of Public Health, Shandong University, 44 West Wenhua Road, Jinan 250012, China; E-Mails: deng.zhengyi@hotmail.com (Z.D.); xun1huan2miao3@yeah.net (H.X.); bjiang@sdu.edu.cn (B.J.); shandawangwei@163.com (W.W.); kangrh@yahoo.com (R.K.); wangxin0730@yeah.net (X.W.); giftatluck@yahoo.com (G.M.); 2 Shandong University Climate Change and Health Center, 44 West Wenhua Road, Jinan 250012, China; 3 National Center for Chronic and Noncommunicable Disease Control and Prevention, Chinese Center for Disease Control and Prevention, 155 Changbai Road, Changping District, Beijing 102206, China; E-Mail: maigengzhou@126.com; 4 National Center for Public Health Surveillance and Information Services, Chinese Center for Disease Control and Prevention, 155 Changbai Road, Changping District, Beijing 102206, China; E-Mails: wsw002@126.com (S.W.); guoqing@chinacdc.cn (Q.G.)

**Keywords:** tropical cyclone, precipitation, infectious diarrhea, bacillary dysentery, risk factor

## Abstract

*Background*: Zhejiang Province, located in southeastern China, is frequently hit by tropical cyclones. This study quantified the associations between infectious diarrhea and the seven tropical cyclones that landed in Zhejiang from 2005–2011 to assess the impacts of the accompanying precipitation on the studied diseases. *Method*: A unidirectional case-crossover study design was used to evaluate the impacts of tropical storms and typhoons on infectious diarrhea. Principal component analysis (PCA) was applied to eliminate multicollinearity. A multivariate logistic regression model was used to estimate the odds ratios (ORs) and the 95% confidence intervals (CIs). *Results*: For all typhoons studied, the greatest impacts on bacillary dysentery and other infectious diarrhea were identified on lag 6 days (OR = 2.30, 95% CI: 1.81–2.93) and lag 5 days (OR = 3.56, 95% CI: 2.98–4.25), respectively. For all tropical storms, impacts on these diseases were highest on lag 2 days (OR = 2.47, 95% CI: 1.41–4.33) and lag 6 days (OR = 2.46, 95% CI: 1.69–3.56), respectively. The tropical cyclone precipitation was a risk factor for both bacillary dysentery and other infectious diarrhea when daily precipitation reached 25 mm and 50 mm with the largest OR = 3.25 (95% CI: 1.45–7.27) and OR = 3.05 (95% CI: 2.20–4.23), respectively. *Conclusions*: Both typhoons and tropical storms could contribute to an increase in risk of bacillary dysentery and other infectious diarrhea in Zhejiang. Tropical cyclone precipitation may also be a risk factor for these diseases when it reaches or is above 25 mm and 50 mm, respectively. Public health preventive and intervention measures should consider the adverse health impacts from tropical cyclones.

## 1. Introduction

Tropical cyclones, also called typhoons, hurricanes or cyclones depending on the geographical area, are periodically associated with disasters [1]. According to the Chinese national standard, tropical cyclones are categorized as tropical depressions when the maximum average wind velocity near the bottom of the tropical cyclone center reaches 10.8 m/s–17.1 m/s; tropical storms at 17.2 m/s–24.4 m/s; severe tropical storms at 24.5 m/s–32.6 m/s; typhoons at 32.7 m/s–41.4 m/s, severe typhoons at 41.5 m/s–50.9 m/s, and super typhoons at 51.0 m/s [2]. Around the world, the annual average number of meteorological disasters including tropical cyclones during the 2002–2011 period was 102. In 2012 they represented 25% of all disasters [3]. Zhejiang Province is one of the most developed provinces in China. It is located at the southeastern coast and hence vulnerable to the tropical cyclones which land on Zhejiang every year, often from July to September, leading to severe injury and large economic losses. Tropical cyclones can cause severe public health consequences, such as death, injuries, psychosocial problems, and increases in infectious diseases [1]. For instance, the super typhoon Saomai in 2006 affected an estimated disaster area of 103,000 hectares, nearly 3.5 million citizens were affected with 1 million forced to evacuate, 204 people died, and 50 thousand buildings were damaged in Zhejiang. The total estimated economic damage in Zhejiang was higher than 12 billion Yuan [4].

Previous studies have analyzed the relationship between tropical cyclones and infectious diseases such as leptospirosis, dengue, cholera, and other infectious diarrhea [5,6,7,8]. For example, typhoon Morakot brought unusual epidemics of leptospirosis to Taiwan in 2009 [9]. Hsieh’s study illustrated that Hurricane Michelle potentially facilitated the spread of the dengue serotype DENV-3 in Havana [8]. Research on infectious diarrhea indicates that tropical cyclones may increase the risk of infectious diarrheal diseases in affected areas. For example, after Cyclone Aila in 2009, there was an increase in infectious diarrheal patients in Bengal, India [10]. An outbreak of norovirus gastroenteritis was detected among evacuees after the landfall of Hurricane Katrina in America in 2005 [11]. In South Korea, there was a significant increase in infectious diarrhea hospitalization cases after a typhoon [7]. However, analyses of these studies were not comprehensive with respect to classification of tropical cyclone and some were not quantitative. In addition, there are few systematical studies conducted in China, which may be quite different from other countries due to variance in local environmental and socio-economic situations.

Infectious diarrhea is a severe infectious disease and an important public health issue in China, leading to more than 7 million people affected each year. In 2009 and 2010, 655,658 and 746,551 cases of infectious diarrhea were reported, respectively [12]. Therefore, there is a need to examine the influence of tropical cyclones on this group of infectious diseases.

Water quality and sanitation coverage are the most important intermediate factors determining the association between tropical cyclones and infectious diarrhea [13]. Tropical cyclones can bring strong precipitation, increasing the turbidity levels of drinking water [14]. Floodwaters caused by extreme precipitation aids in the dispersion of diarrheal-causing pathogens (bacteria, protozoa and viruses) and human infection [15]. Sewage overflow and damage of sewage system due to cyclone precipitation can contaminate clean drinking water sources [16]. Because infectious diarrhea is spread through fecal-oral pathways, contaminated water plays a crucial role in transmission. Therefore, precipitation can be a major influential factor on infectious diarrhea.

Climate extremes including tropical cyclones are projected to increase in China [17]. Precipitation and wind velocity are the typical meteorological characteristics used to describe tropical cyclones. Studying the health effects of these factors can further understand the influence of tropical cyclones on population health. Because tropical cyclones are categorized by wind velocity, analyzing the impact of cyclone categories on infectious diarrhea may reflect the effect of wind velocity. There are few studies on the association between other potentially hazardous factors, such as the typhoon-accompanied precipitation, and infectious diarrhea.

The aim of this study was to quantitatively assess the impact of different classifications of tropical cyclones landing on Zhejiang Province and the varied grades of accompanied precipitation on infectious diarrhea from 2005–2011. Results provide scientific information for establishing a disease alerting system during tropical cyclones. On the basis of the tropical cyclone category and the grade of precipitation, the government can evaluate the risk of infectious diarrhea, and thus, take effective and timely preventive measures.

## 2. Materials and Methods

### 2.1. Study Areas and Demographical Information

**Figure 1:**
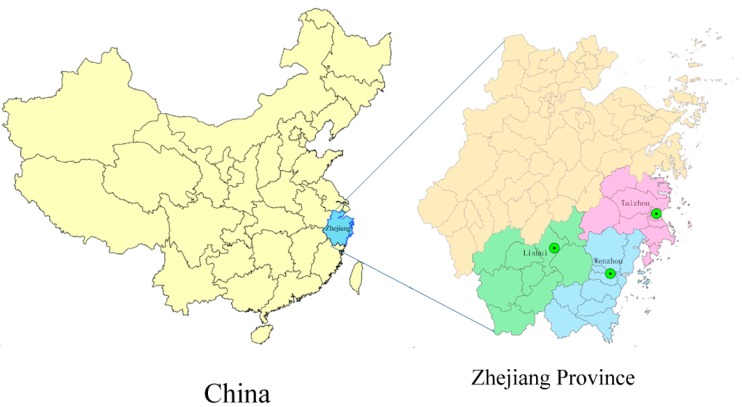
Landing areas of tropical cyclones in Zhejiang Province, China (2005–2011).

From 2005 to 2011, tropical cyclones affected Zhejiang Province, landing primarily in Wenzhou, Taizhou and Lishui. These cities are located in the southeast of Zhejiang Province (Figure 1). The total estimated area of these three cities was 38,495 square kilometers, accounting for 36.97% of Zhejiang Province, with a total population in 2011 of approximately 16,464,800, including 8,508,000 men and 7,956,800 women [18].

### 2.2. Disease Data

Report forms of notified cases of infectious diarrhea in the study areas in Zhejiang Province from 2005 to 2011 were obtained from the National Notifiable Disease Surveillance System (NDSS). The NDSS defines infectious diarrhea as a group of human diseases mainly caused by microbes (including bacteria, parasites, and viruses) that have diarrhea as the typical symptom. Diarrheal diseases include cholera, typhoid, paratyphoid, dysentery (bacillary dysentery and amebic dysentery) and other infectious diarrhea. The category of other infectious diarrhea includes all kinds of infectious diarrhea except cholera, dysentery, typhoid and paratyphoid; examples are salmonella enteritis and rotavirus enteritis. Among these, vibrio para-haemolyticus and salmonella accounted for 65.26% and 20.70% of pathogens in reported bacterial diarrhea. In 2008, the percentage of rotavirus and adenovirus in all reported viral diarrhea was 92.75% and 4.34%, respectively [19]. There were no or few cases of cholera, amebic dysentery, typhoid, and paratyphoid during the study period, thus the analyses focused on bacillary dysentery and other infectious diarrhea. For each case, information on age, gender, date of onset, classification of disease, and location of disease occurrence were obtained from the NDSS records. Cases of bacillary dysentery were diagnosed by physicians, based on the history of exposure, clinical manifestation, and laboratory testing. Symptoms often include abdominal pain, diarrhea, tenesmus, *etc.* Increase in white blood cell counts (WBC ≥ 15/HPF 400 times) and isolation of Shigella from patients’ feces help make a definite diagnosis [20]. All other infectious diarrhea cases were diagnosed by physicians based on the joint evidence from the history of exposure, clinical symptoms, and laboratory test results [21]. The National Communicable Disease Control Act requires physicians in hospitals to report cases of infectious diarrhea to the local center for disease control and prevention within 24 h.

### 2.3. Meteorological Data

Our study included all tropical cyclones that landed on Zhejiang Province from 2005 to 2011. For analytic stability, severe typhoons and super typhoon were combined into one typhoon category. We didn’t include tropical depressions, severe tropical storms, and typhoons due to a lack of tropical cyclone events. Daily meteorological data from 2005 to 2011 of Zhejiang Province were obtained from the China Meteorological Data Sharing Service System (http://cdc.cma.gov.cn/). The meteorological variables included daily average temperature (AT), daily minimum temperature (MiT), daily maximum temperature (MaT), daily average relative humidity (ARH), daily minimum relative humidity (MiRH), daily average air pressure (AAP), daily minimum air pressure (MiAP), daily maximum air pressure (MaAP), daily average wind velocity (AWV), daily maximum wind velocity (MaWV), daily extreme wind velocity (EWV), daily precipitation (DP), daily average vapor pressure (AVP), and daily sunshine duration (SD). We used meteorological data from the meteorological station in the landing city or the nearest station if there was no station in the city of landfall. Based on the classification of precipitation by the China Meteorological Administration and data during the studied tropical cyclones, daily precipitation was categorized as grade 0 (less than 10 mm), grade 1 (10–24.9 mm), grade 2 (25–49.9 mm), grade 3 (50–99.9 mm) and grade 4 (more than 100 mm) [22].

### 2.4. Epidemiological Method and Statistical Analysis

In this study, a unidirectional 1:1 case-crossover design combined with conditional logistic regression models were used to analyze the exposure odds for the case period compared with the control period. Case-crossover design is an effective method to measure the influence of transient exposure to acute diseases [23]. As in a matched case-control study, this design compares each case’s exposure before a case-defining event with that case’s exposure during other period. Because this design is similar to a crossover study, in which each case serves as their own control, characteristics that did not change between case and control periods, such as age, sex, geographic region and so on could not confound the association between tropical cyclones and infectious diarrhea [24]. The case period in this study includes the exposure period to the typhoon and the following maximum incubation period (7 days for bacillary dysentery and 14 days for other infectious diarrheal disease). Control periods were one week for bacillary dysentery and two weeks for other infectious diarrhea before the first onset of case reports. If the estimated date of control day overlapped with a case period, another period one or two prior weeks would be chosen until all control days were not in the case period. Thus, each case day was equally matched with one control day. All of the cases occurring on a given day shared the same exposures in the case and control periods.

Principal component analysis (PCA) was used to reduce the dimensionality of interrelated variables, while retaining the maximum variability in the data [25]. In order to eliminate multicollinearity between meteorological factors, variables except tropical cyclone, DP, MaWV, and EWV were processed using PCA [26]. Principal components (PCs) were calculated and used to represent all other meteorological factors; no multicollinearity between PCs, DP, MaWV and EWV was observed. Based on daily disease cases records, we estimated the odds ratio (OR) and 95% confidence interval (CI) of bacillary dysentery and other infectious diarrhea for different categories of tropical cyclones using the multivariate logistic regression model. In this model, DP, MaWV, EWV and PCs were adjusted. When OR and 95%CI due to exposure to precipitation were calculated, we used grade 0 (0–9.9 mm) as the reference group and adjusted MaWV, EWV, and PCs in the same model. In addition, we calculated the impacts of different precipitation levels on bacillary dysentery and other infectious diarrhea in the two strata: tropical storms and typhoons. The equation of the model is:

(1) $$ln\left(\frac{P}{1-P}\right)=\beta_{0}+\beta_{1}TC+\beta_{2}DP+\beta_{3}EWV+\beta_{4}MaWV+\beta_{5}PC_{1}+\beta_{6}PC_{2}+...+\beta_{m}PC_{n}$$

where *P* is the probability of suffering disease, β_0_ is the intercept, β_1,_ β_2,..._ β_m_ are partial regression coefficients, TC is the category of tropical cyclone, *PC*_1_, *PC*_2,..._ *PC*_n_ are principal components.

Taking into account the incubation period of infectious diarrhea and disease management, we considered lagged effects of lag 0–7 days (e.g., lag 7 means the lagged effect of exposure on the disease at the seventh day after the case period) [27]. All statistical analyses were conducted using SPSS 21.0.

## 3. Results

### 3.1. Basic Information on Tropical Cyclones, Daily Precipitation, and Cases of Infectious Diarrhea during the Study Period

From 2005 to 2011, seven tropical cyclones landed on Zhejiang province, including three tropical storms, three severe typhoons, and one super typhoon. Details of each event are presented in Table 1.

**Table 1:** Basic information of tropical cyclones landing in the study areas from 2005–2011.

Name	Grade (Period)	Landing City	Exposure Duration (d)
Matsa	Severe typhoon (5–6 Aug 2005)	Taizhou	2
Khanun	Severe typhoon (11 Sep 2005)	Taizhou	1
Saomai	Super typhoon (10–11 Aug 2006)	Wenzhou	2
Wipha	Severe typhoon (18–19 Sep 2007)	Wenzhou	2
Kalmaegi	Tropical storm (19 Jul 2008)	Wenzhou	1
Morakot	Tropical storm (8–10 Aug 2009)	Wenzhou	3
Meranti	Tropical storm (11 Sep 2010)	Lishui	1

**Figure 2:**
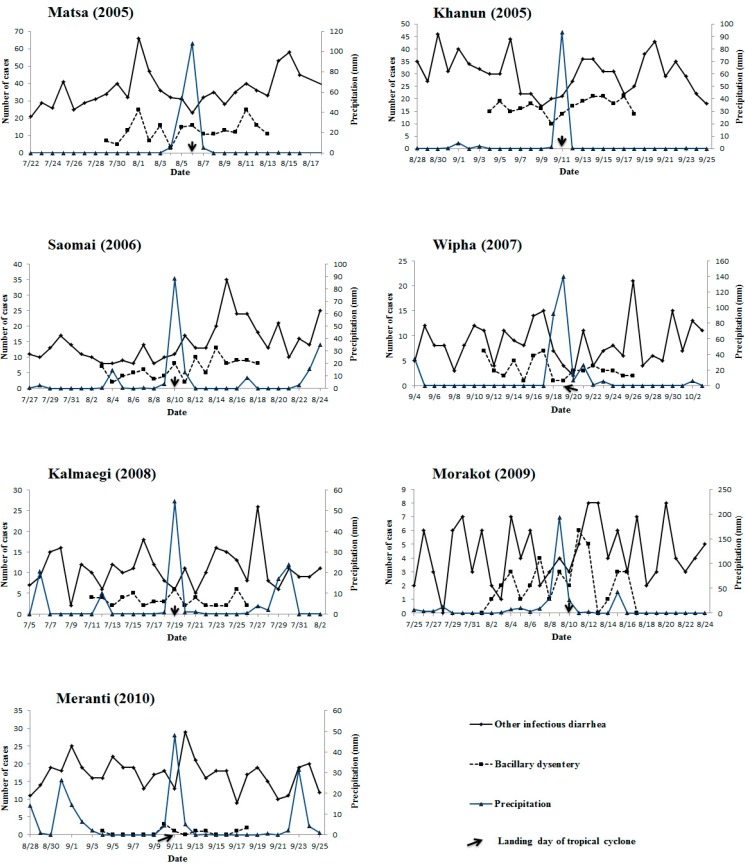
Daily precipitation and infectious diarrhea cases in study period of each tropical cyclone landing on Zhejiang Province during 2005–2011.

Daily precipitation during the study period in the landing city is described in Figure 2. The number of cases of bacillary dysentery and other infectious diarrhea in the landing city are also listed in Figure 2. Description and analyses of the impacts on cholera, amebic dysentery, typhoid, and paratyphoid during the study period were not included due to limited numbers of cases.

### 3.2. Effects of Tropical Cyclones on Infectious Diarrhea (Figure 3)

**Figure 3:**
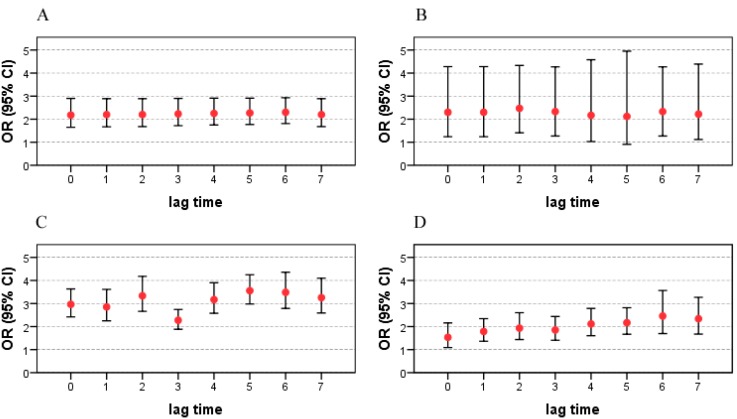
ORs and 95% CIs of tropical cyclones on the risk of bacillary dysentery and other infectious diarrhea on different lagged days. OR: odds ratio; CI: confidence interval; A: impact of typhoon on bacillary dysentery; B: impact of tropical storm on bacillary dysentery; C: impact of typhoon on other infectious diarrhea; D: impact of tropical storm on other infectious diarrhea.

The results indicate that typhoons and tropical storms increased the risk of bacillary dysentery and other infectious diarrhea. The largest impacts of tropical storms and typhoons on bacillary dysentery were on lag 2 days (OR = 2.47, 95% CI: 1.41–4.33) and lag 6 days (OR = 2.30, 95% CI: 1.81–2.93), respectively. The impacts of typhoons on other infectious diarrhea was higher than the effects of tropical storms, with OR = 3.56 (95% CI: 2.98–4.25) on lag 5 days and OR = 2.46 (95% CI: 1.69–3.56) on lag 6 days. More details are shown in Figure 3.

### 3.3. Effects of Precipitation on Infectious Diarrhea

**Figure 4:**
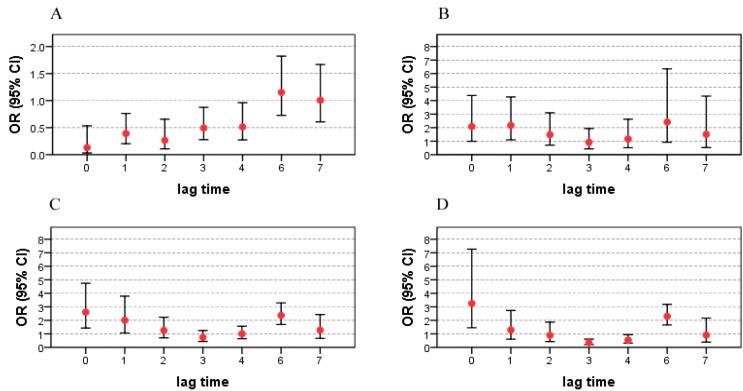
ORs and 95% CIs of tropical cyclone precipitation on the risk of bacillary dysentery on different lagged days. OR: odds ratio; CI: confidence interval; A: impact of grade 1 (10–24.9 mm) precipitation on bacillary dysentery; B: impact of grade 2 (25–49.9 mm) precipitation on bacillary dysentery; C: impact of grade 3 (50–99.9 mm) precipitation on bacillary dysentery; D: impact of grade 4 (more than 100 mm) precipitation on bacillary dysentery.

**Figure 5:**
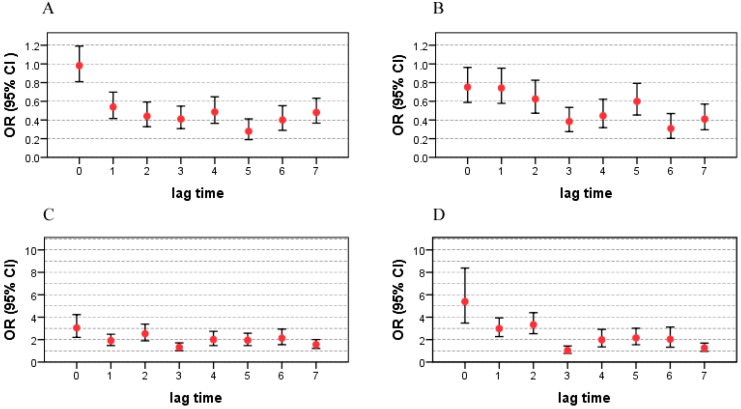
ORs and 95% CIs of tropical cyclone precipitation as the risk for other infectious diarrhea on different lagged days. OR: odds ratio; CI: confidence interval; A: impact of grade 1 (10–24.9 mm) precipitation on other infectious diarrhea; B: impact of grade 2 (25–49.9 mm) precipitation on other infectious diarrhea; C: impact of grade 3 (50–99.9 mm) precipitation on other infectious diarrhea; D: impact of grade 4 (more than 100 mm) precipitation on other infectious diarrhea.

The ORs of different grades of tropical cyclone precipitation on bacillary dysentery are detailed in Figure 4. Tropical cyclone precipitation within 10–24.9 mm (grade 1) was protective with an OR = 0.13 (95% CI: 0.03–0.53) on lag 0 day. However, the tropical cyclone precipitation was a risk factor when daily precipitation reached 25mm and more with the largest OR = 2.17 (95% CI: 1.10–4.27) for grade 2 on lag 1 days, OR = 2.60 (95% CI: 1.43–4.74) for grade 3 on lag 0 day, and OR = 3.25 (95% CI: 1.45–7.27) for grade 4 on lag 0 day. Precipitation grades from 2 to 4 had the largest impacts on bacillary dysentery. Precipitation on lag 5 days was not statistically significant and therefore excluded from the model. Compared with bacillary dysentery, grade 1 and grade 2 precipitation had longer lags with other infectious diarrhea (Figure 5). Tropical cyclone precipitation less than 50 mm was protective of other infectious diarrhea, with OR = 0.28 (95% CI: 0.19–0.41) and OR = 0.31 (95% CI: 0.20–0.47) on lag 5 and lag 6 days, respectively. Precipitation more than 50 mm was a risk factor for other infectious diarrhea. The largest impacts (OR = 3.05, 95%CI: 2.20–4.23 in grade 3; OR = 5.40, 95%CI: 3.47–8.38 in grade 4) were on the days when tropical cyclones landed (lag 0 day). The impact of tropical cyclone precipitation at grade 4 was larger than that of grade 3.

## 4. Discussion

This study quantified the relationships between tropical cyclones from 2005–2011 and infectious diarrhea in Zhejiang Province, China. Our results suggest that typhoon and tropical storm could contribute to an increased risk of bacillary dysentery and other infectious diarrhea.

Increases in diarrheal diseases following tropical cyclone have been reported in developed and developing countries. For instance, a significant increased incidence of diarrhea after typhoon AILA was reported in two Indian subdivisions: Haldia and Egra in 2009 [28]. A Burmese study also observed an obvious increase in the incidence rate for acute diarrhea in the post-Nargis months of 2008 [29]. In South Korea, risk of infectious diarrhea increased more than sixteen percent during typhoon periods [7]. The U.S. Centers for Disease Control and Prevention (CDC) reported that gastrointestinal diseases were the most commonly recorded acute disease among evacuees from Memphis and Tennessee after hurricane Katrina in 2005 [30]. Results from these studies are consistent with our findings in that there is a relationship between tropical cyclones and increase in diarrhea in Zhejiang.

Our study also identified that tropical storms and typhoons differentially influenced different groups of infectious diarrhea. Both tropical storms and typhoons had lagged effects, ranging from 2 to 6 days. For the group of other infectious diarrhea, the impact of typhoons was apparently higher than that of tropical storms. Whereas for bacillary dysentery, risks of typhoons were slightly lower than that of tropical storms. However, there were merely minimal differences between the ORs of these types of cyclones, thus we cannot draw a definitive conclusion that storms and typhoons influence bacillary dysentery to different extents. This may be due to the limited number of tropical cyclone related events analyzed. In addition, because the group of other infectious diarrhea includes several kinds of infectious diarrhea, the different causal mechanisms for each disease category may have influenced the result. Because the categorization of tropical cyclones is defined by wind velocity, the impact of a particular category of tropical cyclone can represent the influence of wind speed associated with it. Our study suggests that stronger wind might increase the risk for the group of other infectious diarrhea. Results from previous research were inconsistent in how wind speed could impact infectious diarrhea. A study suggested increased wind speed could result in a lower prevalence of bacillary dysentery [31]. However, studies in Shanghai, Wuhan and northeastern China found no significant relationships between wind speed and infectious diarrhea [32,33,34].

We also identified that cyclone precipitation could be either a protective or a risk factor for the occurrence of infectious diarrheal diseases, depending on the amount of precipitation. Twenty-five millimeter and 50 mm could be turning points of the impacts of tropical cyclone precipitation on bacillary dysentery and other infectious diarrhea, respectively. When the daily precipitation was higher than 25 mm or 50 mm, *i.e.*, the greater the tropical cyclone precipitation, the higher the risk of suffering bacillary dysentery or other infectious diarrhea. We found that ORs of precipitation between 10–25 mm were significantly lower than one, which indicated that tropical cyclone precipitation between 10–25 mm was probably beneficial to human health. The mechanism of this protective effect needs further study. One possible reason is that tropical cyclones are characterized by strong wind and precipitation; thus, tropical cyclones with relatively little precipitation and strong wind result in dry weather, which may be adverse to human health. In addition, a very short or no lagged effect was observed for tropical cyclone precipitation. These results suggest that more attention should be focused on prevention and control of infectious diarrhea diseases when precipitation reaches 25 mm or above following tropical cyclones. Previous studies documented that heavy precipitation was associated with gastrointestinal illness. Positive association between extreme precipitation and gastrointestinal illness-related hospital admission also was reported from 2004 to 2007 in Chennai, India [35]. Our findings indicate that risks of bacillary dysentery increased significantly after exposure to heavy rain (25.0–49.9 mm), torrential rain (50–99.9 mm), and extreme torrential rain (≥100 mm) caused by tropical cyclones, which is similar to the Taiwanese study, which found that risks of bacillary dysentery increased nearly twofold after exposure to torrential rain and seven times after exposure to extreme torrential rain [27]. Also in northeast China, a positive correlation between precipitation and bacillary dysentery was noted [36].

However, the association between precipitation and diarrhea was far from clear. In a study in Wuhan, China, researchers obtained results confirming an inverse association between precipitation and infectious diarrhea [32]. Another study in Ecuador found that heavy precipitation events were associated with increased diarrheal disease incidence following dry periods and decreased diarrhea incidence following wet periods [37]. Another two studies did not detect any significant effects of precipitation on infectious diarrhea in cities at the northern and southern parts of China [38,39]. This observed discrepancy may have resulted from socioeconomic factors such as individual perceptions and behaviors, socio-economic status, and access to health services. For instance, citizens’ low awareness of health issues and unhealthy drinking habits may make them more susceptible to infectious diarrhea. Economic status determines the level of health service and the drinking water sanitation to some extent, and thus, may influence the risk of suffering infectious diarrhea. In addition, different intensities of precipitation in different studies might be another reason for varying results. For example, the Taiwanese study investigated the impact of extreme torrential precipitation (>350 mm/day) on bacillary dysentery [27], while the impact of a quite lower daily precipitation (3.1 mm) was studied in Wuhan [32].

Due to limited number of tropical cyclone events in Zhejiang province during 2005–2011, the impacts of other categories of tropical cyclones, such as tropical depression and severe tropical storm, on infectious diarrhea with longer lags could not be assessed in our study. In addition, there might be other factors that could influence transmission of infectious diarrhea.

Irrespective of these limitations, there are some advantages of this study. First, the case-crossover study design, similar to a matched case–control study, is advantageous in controlling confounders. Confounders, such as geographical characteristics, behaviors and economic status, would remain constant across the study period. Second, we considered the day of week effect in the selection of the control period for controlling any possible time trend. Third, we had a relatively long study period with quality data collected from patients’ case records. In particular, we calculated the impacts of seven different tropical cyclones on bacillary dysentery and other infectious diarrhea, producing robust results.

## 5. Conclusions

Our study shows that both tropical storm and typhoon could increase the risk of bacillary dysentery and other infectious diarrhea in the study areas in China. Tropical cyclone precipitation is a risk factor for bacillary dysentery and other infectious diarrhea when it reaches 25 mm and 50 mm or above. These findings may assist in public health preparation to reduce the adverse health impacts from tropical cyclones.
